# Preliminary report of *Mycoplasma Wenoynii* and *Candidatus Mycoplasma haemobos* infection in Korean native cattle

**DOI:** 10.1186/s12917-024-03976-2

**Published:** 2024-03-26

**Authors:** Youngjun Kim, Hannah Kim, Jae-Hyeon Choi, Hyung-Chul Cho, Min-Jeong Ji, Yu-Jin Park, Jinho Park, Kyoung-Seong Choi

**Affiliations:** 1 Department of Animal Hospital, Genetic Improvement Center, National Agricultural Cooperative Federation, Hanwoo, Seosan, 31948 Republic of Korea; 2 Department of Veterinary Internal Medicine, College of Veterinary Medicine, Jeonbuk University, Iksan, 54596 Republic of Korea; 3 College of Arts and Sciences, University of Washington, Seattle, WA 98195 USA; 4 Department of Horse/Companion and Wild Animals, College of Ecology and Environmental Science, Kyungpook National University, Sangju, 37224 Republic of Korea; 5 Department of Animal Science and Biotechnology, College of Ecology and Environmental Science, Kyungpook National University, Sangju, 37224 Republic of Korea

**Keywords:** *Mycoplasma wenyonii*, *Candidatus* Mycoplasma haemobos, Korean native cattle, Grazing, anemia

## Abstract

**Background:**

Hemotropic mycoplasmas or hemoplasmas are bacteria that attach to the erythrocyte surface and cause bovine hemoplasmosis. Two species, *Mycoplasma wenyonii* and *Candidatus* Mycoplasma haemobos, have been identified and shown to be distributed worldwide. However, there is currently no information available on hemoplasmas in cattle in the Republic of Korea. The aim of this study was to investigate the presence of hemoplasmas in Korean native cattle and to evaluate the association between hemoplasma infection and anemia.

**Methods:**

One farm was selected, at which blood samples were collected from 104 Korean native cattle [grazing cattle (*n* = 89) and housed cattle (*n* = 15)]. Hemoplasmas were detected via polymerase chain reaction analysis and complete blood counts were also performed.

**Results:**

The overall prevalence of hemoplasmas was 34% (35/104); 20.2% (21/104) for M. *wenyonii*, 3.8% (4/104) for *C.* M. haemobos, and 9.6% (10/104) for co-infection. *Candidatus* Mycoplasma haemobos was detected only in grazing cattle. Of red blood cell (RBC) parameters, *C.* M. haemobos-infected cattle had lower RBC and hematocrit, and higher mean cell volume than hemoplasma-negative cattle, although none of these differences were statistically significant. This is the first study to report the occurrence of *M. wenyonii* and *C.* M. haemobos. *Mycoplasma wenyonii* is more prevalent than *C.* M. haemobos in Korean native cattle. The results did not show an association between hemoplasma infection and anemia.

**Conclusions:**

Considering the infection rate of hemoplasmas shown in this study, further studies, such as on the pathogenicity and clinical significance of hemoplasmas are necessary.

**Supplementary Information:**

The online version contains supplementary material available at 10.1186/s12917-024-03976-2.

## Background

The climate of the Korean Peninsula is rapidly becoming subtropical, and warmer temperatures have already resulted in accelerated parasitic development and an extreme rise in vector populations [1]. These climatic changes have a widespread impact on the ecosystem. The temporal and spatial changes in temperature, precipitation, and humidity that occur under different climate conditions affect the biology and ecology of vectors and intermediate hosts, consequently increasing the risk of disease transmission [2]. Arthropods are also closely linked to the climate, and there is growing concern about the increasing prevalence of vector-borne diseases (VBDs) on the Korean peninsula. VBDs in particular have been reported to cause serious health problems in ruminants, leading to substantial economic losses to the livestock industry worldwide [3].

Hemotropic mycoplasmas or hemoplasmas are epi-erythrocytic bacteria that infect a wide variety of animals including humans [4–6]. Their infections typically remain subclinical, but may lead to more severe diseases, such as hemolytic anemia, production loss, and infertility [7–10], depending on the animal species [11]. To date, two major hemoplasmas have been identified in cattle: *Mycoplasma wenyonii* and *Candidatus* Mycoplasma haemobos [12–14]. *Mycoplasam wenyonii* was first described in a splenectomized calf in 1934 [12] and *C.* M. haemobos has been reported more recently due to the development of molecular methods. Infected cattle exhibit anemia, pyrexia, hemoglobinuria, lymphadenopathy, edema of the scrotum and hind limbs, swollen teats, and decreased milk production [13, 15, 16]. Some studies have shown that hemoplasmas may be associated with regenerative anemia or changes of various blood parameters [8, 9, 17, 18]. Nevertheless, the pathogenicity of hemoplasmas in cattle is still unclear. The possible transmission routes of these species may involve arthropods, such as ticks, fleas, flies, and mosquitoes [19, 20], or occur via direct contact with contaminated blood [21]. Moreover, there are reports of transplacental transmission during pregnancy [18, 22, 23].

Hemoplasma infections have been shown to be distributed worldwide, with varying prevalence from country to country [4, 18, 21, 24–29]. However, to the best of our knowledge, no reports of hemoplasmas in cattle in the Republic of Korea (ROK) have been published. The recent rapidly changing climate of the ROK could provide the optimal conditions for tick expansion, and the frequency of occurrence of various VBDs could increase significantly. Therefore, the present study was established to investigate the presence of hemoplasmas that had not been reported previously in the ROK, to compare the prevalence in Korean native cattle managed on pasture and indoors, and to evaluate the association between hemoplasma infection and anemia in grazing cattle.

## Methods

### Sampling

**Fig. 1:**
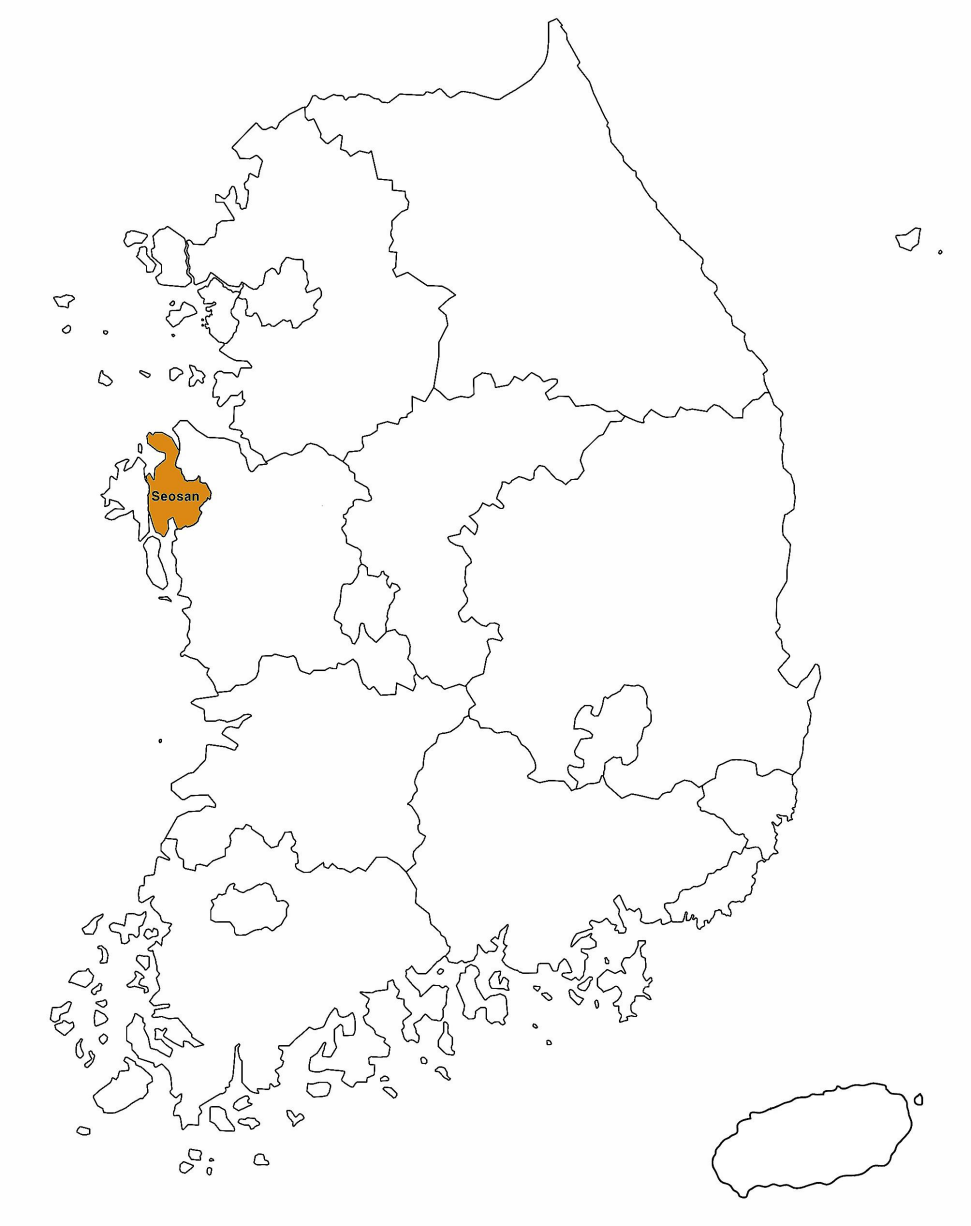
Map showing the region where blood samples were collected in the Republic of Korea

A total of 104 blood samples were collected on one farm in the ROK (Fig. 1). Eighty-nine cattle were allowed to graze on pastureland, whereas 15 cattle were raised indoors. Blood samples of Korean native cattle were collected from jugular vein in tubes containing EDTA (BD Vacutainer^®^; Beckton Dickinson Vacutainer Systems, Franklin Lakes, NJ, USA) and were immediately delivered to the laboratory on the day of blood collection. Hematological examinations included red blood cell (RBC) profile comprising RBC count, hematocrit (HCT), mean cell volume (MCV), mean corpuscular hemoglobin concentration (MCHC), and white blood cell (WBC) count. The samples were processed using an automatic blood analyzer (IDEXX Procyte Dx; IDEXX Laboratories, Westbrook, ME, USA). These samples were used to compare the hematological changes in hemoplasmas infection according to growth type: grazing and barn feeding system. The cattle used in this study were all female, 22−35 months old, and clinically healthy.

### DNA extraction, polymerase chain reaction, and sequencing analysis

DNA was extracted from 200 µL of blood using DNeasy Blood Kit (Qiagen, Hilden, Germany), in accordance with the manufacturer’s instructions, and stored at −80 °C until analysis. Detection of hemoplasmas was conducted using a multiplex-PCR method as previously described [30]. PCR was performed under the following conditions: 94 °C for 5 min; followed by 35 cycles of 1 min at 94 °C, 59 °C for 1 min, and 72 °C for 1 min; and then final extension at 72 °C for 5 min. Distilled water was used as a negative control in all PCRs. The amplicons were separated by agarose gel electrophoresis, visualized through staining with ethidium bromide, and photographed under UV light. PCR products were purified using the Accupower PCR purification Kit (Bioneer, Daejeon, ROK), in accordance with the manufacturer’s instructions and directly sequenced (Bioneer). All of the obtained nucleotide sequences for each pathogen were aligned using BioEdit software and were compared with the reference sequences from the National Center for Biotechnology Information database (http://www.ncbi.nlm.nih.gov) to determine similarity. The nucleotide sequences obtained in this study were assigned the following accession numbers: OR425074−OR425092 for *M. wenyonii* and OR425093−OR425096 for *C.* M. haemobos.

### Statistical analysis

The prevalence rates were calculated with 95% confidence interval (CI). Data were expressed as mean ± standard deviation. Statistical analyses were performed using the SPSS 29.0 software package (SPSS, Chicago, Illinois, USA). The results of the hematological analyses were categorized by each infection. The Shapiro−Wilk test was used to evaluate distribution normality and the Bartlett test for homogeneity of variances. One-way ANOVA was performed to analyze hematological findings. A *P-*value of less than 0.05 was considered statistically significant.

## Results

Of a total of 104 blood samples, the overall infection rate of hemoplasmas was 34% (35/104; 95% CI: 24.6–42.7) in Korean native cattle; 13.3% (2/15) in housed cattle and 37.1% (33/89) in grazing cattle. Among these, *M. wenyonii* and *C.* M. haemobos were detected in 21 (20.2%; 95% CI: 12.5–27.9) and 4 (3.8%; 95% CI: 0.1−7.5) cattle, respectively. Co-infection was found in 10 cattle (9.6%; 95% CI: 3.9−15.3) (Table 1). When comparing the prevalence of hemoplasma species according to grazing types, *M. wenyonii* infection was identified at a rate 3.5-fold higher in grazing cattle (22.5%) than in housed cattle (6.7%), whereas *C.* M. haemobos was found only in grazing cattle (4.5%; Table 1). Moreover, co-infection was detected at a rate 1.5-fold higher in grazing cattle (10.1%) than in housed cattle (6.7%; Table 1). Overall, there were differences in the prevalence of hemoplasmas between housed and grazing cattle. The occurrence of *M. wenyonii* was higher than that of *C.* M. haemobos in grazing cattle.

**Table 1:** Prevalence of hemoplasmas detected in Korean native cattle according to grazing type

Grazing type	Mycoplasma wenyonii		Candidatus M. haemobos		Co-infection	
	No. of positive samples (%)	95% CI	No. of positive samples (%)	95% CI	No. of positive samples (%)	95% CI
Housing (*n* = 15)	1 (6.7%)	0.0–19.3	0 (0%)	0.0–0.0	1 (6.7%)	0.0–19.3
Grazing (*n* = 89)	20 (22.5%)	13.8–31.1	4 (4.5%)	0.2–8.8	9 (10.1%)	3.8–16.4
Total (*n* = 104)	21 (20.2%)	12.5–27.9	4 (3.8%)	0.2–7.5	10 (9.6%)	3.9–15.3

95% CI: confidence interval

Hematological parameters, such as RBC, HCT, MCV, MCHC, and WBC, in grazing cattle were compared between the hemoplasma-infected group and the hemoplasma-negative group. RBC counts were lowest in the *C.* M. haemobos-infected group. HCT values were also decreased in cattle with *C.* M. haemobos infection compared with those in the hemoplasma-negative cattle, but co-infection led to the lowest HCT levels (Table 2). The values of MCV were highest in the *C.* M. haemobos-infected group, whereas they were lowest in cattle with co-infection (Table 2). Interestingly, MCHC values did not differ markedly in all three groups, except for the co-infected group, which showed increased values (Table 2). The WBC counts were highest in cattle with co-infection, followed by the *M. wenyonii*-infected group. There were no differences in WBC counts between the *C.* M. haemobos-infected group and the hemoplasma-negative group (Table 2). Nevertheless, there were no statistically significant differences in all of these values (Table 2). Our results showed lower RBC and HCT values, and higher MCV in *C.* M. haemobos infection.

**Table 2:** Comparison of hematological examination in grazing cattle (*n* = 89) according to hemoplasma infection

Parameters	M. wenyonii infection (*n* = 20)	*C.* M. haemobos infection (*n* = 4)	Co-infection (*n* = 9)	Hemoplasmas negative (*n* = 57)	P-value
RBC (10^6^/µL)	4.90 ± 1.47	4.00 ± 1.22	5.10 ± 0.99	5.00 ± 1.16	0.5252
HCT (%)	27.2 ± 6.48	25.3 ± 7.65	25.2 ± 4.45	27.9 ± 4.49	0.4153
MCV (fL)	58.3 ± 13.07	64.6 ± 7.98	49.7 ± 5.63	57.6 ± 12.00	0.0584
MCHC (g/dL)	31.0 ± 1.65	31.1 ± 1.32	32.6 ± 1.23	31.1 ± 1.61	0.0744
WBC (10^3^/µL)	14.8 ± 2.13	14.6 ± 3.81	15.9 ± 3.37	14.6 ± 3.08	0.7033

RBC, red blood cell; HCT, hematocrit; MCV, mean corpuscular volume; MCHC, mean corpuscular hemoglobin concentration; WBC, white blood cell

Of the 21 *M. wenyonii-* and four *C.* M. haemobos-positive samples, 19 and four sequences were successfully obtained and used for sequencing analysis. Our 19 sequences exhibited 91.79–99.34% identity to each other and shared 91.84–99.67% similarity to those reported from cattle in several countries such as Austria (KY412804), China (FJ375309), Cuba (MG948624), England (DQ641256), Japan (EU367964), and Turkey (OM468183) (Supplementary Table 1). Four *C.* M. haemobos showed 94.68–96.74% identity to each other. These sequences displayed 94.85–98.91% identity to those reported in cattle and 95.71–99.03% to those from water buffalo (Supplementary Table 2). Hemoplasmas circulating in Korean native cattle are closely related to those detected in Turkey.

## Discussion

To the best of our knowledge, this is the first study to report the occurrence of *M. wenyonii* and *C.* M. haemobos in cattle in the ROK. The overall prevalence of hemoplasmas in this study was 33.7%, which was higher than we anticipated. Our results are similar to those from Brazil (34.83%) [6], Uganda (32.2%) [31], and Turkey (31.64%) [29], but were markedly lower than those reported in the Philippines [11], Cuba [21], Germany [18], Japan [26], Nigeria [32], and Switzerland [33]. According to our results, the infection rates of *M. wenyonii* and *C.* M. haemobos were 20.2% and 3.8%, respectively, suggesting that *M. wenyonii* is more prevalent in Korean native cattle than *C. M. haemobos*. The prevalence of *C.* M. haemobos in Korean native cattle was relatively low compared with that in other countries [18, 21, 29, 34–37]. Moreover, in the current study, *C.* M. haemobos was detected only in grazing cattle. This can be explained by the difference of management systems, housed vs. grazing. However, we cannot draw a definitive conclusion at this point because the number of samples from indoor housed cattle was very low. Further research should thus be conducted to determine the prevalence of hemoplasmas in cattle according to management systems via extensive epidemiological survey.

Currently, little is known about bovine hemoplasmas (i.e., vectors and clinical significance) in the ROK. Our results indicate that hemoplasmas infection in cattle may be associated with ticks. This is supported by the present result that the occurrence of hemoplasmas was more frequent in grazing cattle. This is because grazing cattle have a much greater risk of exposure to ticks than housed cattle. Although hemoplasmas were not detected from tick-infested cattle in this study, there have been several reports on hemoplasma detection from tick species [11, 28, 38] and *Rhipicephalus* spp. have been identified as potential vectors of *Mycoplasma* species [11, 20, 39]. However, this tick species has rarely been found in the ROK, raising the possibility that other vectors, such as flies or lice, may transmit hemoplasmas. Several studies have reported the occurrence of *M. wenyonii* in hematophagous flies, suggesting that these flies can transfer the pathogen to new hosts [22, 40]. Moreover, in a previous study, it was noted that *M. wenyonii* is transmitted more by blood-sucking flies than *C.* M. haemobos [22]. In the ROK, flies are the most common ectoparasite around barns and cattle are more likely to attract flies due to their large size and odor emission, leading to them infesting cattle. For this reason, the possibility that hemoplasmas can be transmitted by both ticks and flies cannot be ruled out. Furthermore, it has been reported that *M. wenyonii* could be transmitted by lice [13]. Given the high prevalence of hemoplasmas in the ROK, there is an urgent need to identify the vector that transmits bovine hemoplasmas and establish control strategies to eradicate these vectors.

Our results revealed no significant association between hemoplasma infection and anemia. This is inconsistent with other studies reported previously [8, 9, 17, 18]. In addition, indicators of anemia (RBC and HCT) were generally low in grazing cattle (both hemoplasma-negative and hemoplasma-positive) compared with the reference values. Since grazing cattle are susceptible to ticks, the possibility of infection with other VBDs, such as theileriosis and anaplasmosis, cannot be ruled out in hemoplasmas-negative cattle. The results showed that *C.* M. haemobos-infected cattle had lower RBC and HCT levels but higher MCV values, unlike cattle with *M. wenyonii*. This finding is consistent with that reported in Japan [17, 36]. The low RBC and HCT levels and high MCV values observed in *C.* M. haemobos-infected cattle are associated with regenerative anemia; however, the present results did not provide clues that hemoplasma infection causes anemia because of the limited number of samples. Therefore, further investigations are needed to clarify the relationship between anemia and hemoplasmas infection.

## Conclusions

The present study describes the first molecular detection of hemoplasmas infection in Korean native cattle. Our findings indicate that *M. wenyonii* is prevalent in grazing cattle. Although *C.* M. haemobos-infected cattle showed low RBC and HCT levels and high MCV levels, these results suggest that there is no significant association between hemoplasma infection and anemia. Further studies are required to determine the pathogenicity, clinical significance, and vectors through massive epidemiological investigation.

### Electronic supplementary material

Below is the link to the electronic supplementary material.

Supplementary Material 1

Supplementary Material 2

## Data Availability

All data generated during this study are included in this published article and its additional files. The datasets used and/or analyzed during the current study are available from the corresponding author on reasonable request. Sequences obtained in this study have been submitted to GenBank under accession numbers: OR425074−OR425092 for *M. wenyonii* and OR425093−OR425096 for *C.* M. haemobos.

## Abbreviations

*C.* M. haemobos: *Candidatus* Mycoplasma haemobos
HCT: hematocrit
MCV: mean cell volume
MCHC: mean corpuscular hemoglobin concentration
*M. wenyonii*: *Mycoplasma wenyonii*
PCR: polymerase chain reaction
RBC: red blood cell
ROK: Republic of Korea
WBC: white blood cell
VBDs: vector-borne diseases
